# Supplementary material for: Green accounting and reporting in Bangladesh’s pharmaceutical and textile industries: A holistic perspective
Source: PLoS One. 2024 Sep 10;19(9):e0310236. doi: 10.1371/journal.pone.0310236 (PMC11386424; doi:10.1371/journal.pone.0310236)
Supplement: S2 File — (PDF) [file pone.0310236.s002.pdf]

Appendices:

Table A1: Descriptive analysis of the dependent and independent variables of Textile Companies (all BDT in million)

Variables	N	Minimum	Maximum	Mean	Std. Deviation	Cronbach's Alpha
Energy	110	0.00	1.00	0.32	0.299	0.823
Effluents and Waste	110	0.00	1.00	0.16	0.348	0.823
Products and Services	110	0.00	1.00	0.47	0.264	0.823
Materials	110	0.00	1.00	0.21	0.362	0.823
Supplier Environmental Assessment	110	0.00	1.00	0.22	0.342	0.823
Biodiversity	110	0.00	1.00	0.06	0.228	0.823
Emission	110	0.00	1.00	0.61	0.336	0.823
Water	110	0.00	1.00	0.48	0.337	0.823
Environmental Compliance	110	0.00	1.00	0.33	0.231	0.823
Environmental Grievance Mechanism	110	0.00	1.00	0.03	0.164	0.823
Occupational Health and Safety	109	0.00	0.83	0.46	0.251	0.823
Environmental Accounting and Reporting Practice	110	0.00	0.88	0.30	0.197	0.823
Ownership Form	110	0.00	0.91	0.33	0.206	0.823
Firm Features General Characteristics	110	0.00	1.00	0.66	0.305	
Total Assets	107	214.12	17464.64	4143.06	3589.86	0.777
Total Investment	109	0.00	21933.36	4008.22	3940.40	0.782
Total Share Holder Equity	106	34.22	7444.41	2264.67	2000.91	0.786
Total Debt	107	39.79	19274.21	1860.07	2504.30	0.795
ROA	107	-1.85	14.42	3.64	2.62	0.823
ROE	106	-4.03	94.22	8.77	10.56	0.823
ROI	106	-3.83	23.33	3.71	3.01	0.823
EPS	107	-0.64	62.57	3.74	7.85	0.823
NPR	107	-38.73	1189.36	156.47	200.03	0.820
Stakeholder Pressure	110	0.00	1.00	0.38	0.262	0.823
External Factor	110	0.00	1.00	0.58	0.294	0.823
Source: Source: The Authors, based on statistical analysis						

Table A2: Descriptive analysis of the dependent and independent variables of Pharmaceutical Companies (all BDT in million).

Variables	N	Minimum	Maximum	Mean	Std. Deviation	Cronbach's Alpha
Energy	65	0.00	1.00	0.40	0.330	0.770
Effluents and Waste	65	0.00	1.00	0.22	0.354	0.770
Products and Services	65	0.00	1.00	0.53	0.373	0.770
Materials	65	0.00	1.00	0.29	0.330	0.770

Supplier Environmental Assessment	65	0.10	1.00	0.53	0.350	0.770
Biodiversity	65	0.00	1.00	0.26	0.357	0.770
Emission	65	0.10	1.00	0.39	0.329	0.770
Water	65	0.10	1.00	0.32	0.301	0.770
Environmental Compliance	65	0.00	1.00	0.42	0.227	0.770
Environmental Grievance Mechanism	65	0.00	0.10	0.01	0.029	0.770
Occupational Health and Safety	65	0.08	1.00	0.59	0.284	0.770
Environmental Accounting and Reporting Practices	65	0.06	0.81	0.36	0.196	0.770
Capital Employed	64	-56676.79	108181.25	10489.52	22455.79	0.709
Firm Features General Characteristics	65	0.20	1.00	0.82	0.284	0.770
Total Assets	64	172.80	122746.30	15158.32	23563.15	0.695
Total Investment	64	173.70	128511.71	17476.54	27530.85	0.696
Total Share Holder Equity	64	100.00	125409.00	11684.73	20844.63	0.715
Total Debt	64	67.66	59372.99	5791.81	10507.74	0.731
ROA	64	-14.59	40.79	11.39	11.01	0.770
ROE	64	-0.51	110.05	20.96	22.79	0.770
ROI	64	-0.49	40.79	11.51	10.63	0.770
EPS	63	-52.75	72.72	15.62	21.36	0.770
NPR	64	-635.48	11606.19	1409.27	2467.51	0.765
Stakeholder Pressure	65	0.25	1.00	0.52	0.251	0.770
External Factor	65	0.00	1.00	0.86	0.253	0.770
Source: The Authors, based on statistical analysis						

Table A3: Evaluation Matrix

Scale 0: No disclosure, this topic is not mentioned in the report

Scale 1: The topic is mentioned in the annual reports and disclosure made.

**GRI Guidelines**	**Variable code**	**Environmental Accounting Reporting Practice disclosure**
GRI 302	EARP1	Energy level practice
GRI 306	EARP 2	Effluents and waste management
GRI 3 and 301	EARP 3	Product and service materials for organization
GRI 308	EARP 4	Supplier environmental assessment practice
GRI 304	EARP 5	Biodiversity maintained
GRI 305	EARP 6	Emission disclosure ratio
GRI 303	EARP 7	Water management system

GRI 1	EARP 8	Environmental compliance
GRI 406	EARP 9	Grievance mechanism
**Firm Features**		
GRI 201	GC 1	Firm size
GRI 201	GC 2	Category
GRI 202	GC 3	Organizational culture
GRI 1	GC 4	Corporate social responsibility
GRI 206	GC 5	Position in the value chain
GRI 404	GC 6	Strategic attitudes
GRI 402	GC 7	Motivation of managers attitude
GRI 410	GC 8	Risk management
GRI 02	GC 9	Corporate governance
**Stakeholder Pressure**		
GRI 1	SP 1	Organizational Regulation (section 2)
GRI 3	SP 2	Media
GRI 3	SP 3	Pressure from Employees
GRI 1	SP 4	Pressure from Investors
**External Factors**		
GRI 414	EF 1	Social environmental Impact
GRI 3	EF 2	Production process
GRI 201	EF 3	Firm Location
GRI 202	EF 4	Global Culture
N. B : Rest Numeric data for related variables are collected from the reports		

Source: Authors compilation based on GRI Guidelines

Table A4: Variables Item Sources

Category	Sources
**Firm Features:**<br>Firm size<br>Category status<br>Organizational culture<br>Corporate Social Responsibilities<br>Position in the Value Chain<br>Strategic Attitudes<br>Motivation of Managers Attitudes<br>Risk Management<br>Financial Resources<br>Firms Performance	Karimi et al., 2017; Chang & Deegan, 2010; Qian et al., 2011; Frost & Wilmshurst, 2000; Hackston & Milne, 1996; Frost & Seamer, 2002, Ribeiro et al., 2010; Christ & Burritt, 2013; Muttakin & Subramaniam, 2015; Nguyen et al., 2017; Sulaiman et al., 2014; Welbeck et al., 2017; Andrew et al., 2006; Ribeiro & Aibar-Guzman, 2010; Frost and Toh, 1998a.
**Stakeholder Pressure:**<br>Organizational Regulation<br>Media<br>Pressure from Employees<br>Pressure from Investors	Chang & Deegan, 2010; Bennett et al. 2011; De Villiers & Staden, 2010; Deegan & Rankin, 1997;

**External factors:**<br>Social Environmental Impact<br>Firms Location<br>Global Culture<br>Regulatory Environment<br>Production Process	Karimi et al., 2017; Chang & Deegan, 2010; Qian et al., 2011; Christ & Burritt 2013; Ferreira et al., 2010; McElroy et al., 2005

Source: Authors compilation based on secondary literature review
